# Corrigendum

**DOI:** 10.3402/gha.v7.25010

**Published:** 2014-06-11

Regarding the paper titled: ‘Climate change threats to population health and well-being: the imperative of protective solutions that will last’ *by Tord Kjellstrom and Anthony J. McMichael*.

Published in *Global Health Action* (section “Global Health Beyond 2015”) 3 April 2013. Citation: Glob Health Action 2013, **6**: 20816 - http://dx.doi.org/10.3402/gha.v6i0.20816

The legend for Table 1 is incorrect:

It currently reads: “Currency=millions of USD PPP; GHG=greenhouse gases.”

This sentence should read: “Currency=billions of USD PPP; GHG=greenhouse gases.”

